# Occupational stress and the importance of self-care and resilience: focus on veterinary nursing

**DOI:** 10.1186/s13620-017-0108-7

**Published:** 2017-09-25

**Authors:** Ciaran Lloyd, Deirdre P. Campion

**Affiliations:** UCD School of Veterinary Medicine, University College Dublin, Belfield, D04 W6F6, Dublin, Ireland

**Keywords:** Veterinary nursing, Occupational stress, Burnout, Compassion fatigue, Psychological resilience

## Abstract

**Background:**

Burnout and compassion fatigue are frequently mentioned in relation to veterinary work. Veterinary nursing is a caring profession and those who seek a career within this field do so because of a natural empathetic desire to care for animals. However it is the individuals who are the most caring and empathetic towards others that will be most at risk of experiencing occupational stress when they are confronted with psychologically demanding workplace roles and working environments.

**Main body:**

Burnout is considered an ‘unintentional end point’ for certain individuals who are exposed to chronic stress within their working environment. When suffering burnout, a person may experience emotional exhaustion, may become more cynical or they may have a reduced sense of personal accomplishment in regards to their own work. Signs of burnout can include increased levels of absenteeism at work, or the working standards of that staff member may decline below that of what would normally be expected of them. This could directly impact on patient care in the veterinary practice.

Working in a role that places emotional demands on staff, such as a need to show compassion and empathy towards clients who are emotionally distressed, puts staff at risk from experiencing compassion fatigue. Workplace supports may include appropriate debriefing sessions among willing participants, particularly after an emotionally stressful encounter with a client.

Taking personal responsibility for care of one’s own mental and physical health is just as important as taking care of the patient’s health. Personal strategies may include lifestyle changes, adopting a healthier lifestyle, reduction of working hours, and ensuring adequate sleep. Adopting healthy self-care strategies can promote characteristics of "resilience" - personal qualities or traits such as optimism, self-confidence, level headedness, hardiness, and having the ability to be resourceful during times of adversity.

**Conclusion:**

All veterinary staff may be better prepared to deal with occupational stress related conditions if they gain better insight and ability to recognise the condition in self and others, and if provided with the toolkits to develop coping strategies and resilience.

## Background

Psychosocial risks are a particular form of workplace hazard. According to the European Agency for Safety and Health at Work, psychosocial risk may result in work-related stress, burnout and depression [1]. An increasingly accepted definition of workplace stress, as described in a report commissioned by that Agency, is as “a psychological state which is part of and reflects a wider process of interaction between the person and their work environment” [2].

Workplace and occupational stress is an area that has drawn a great deal of attention for human health care workers over the years, but not until relatively recently has it been reviewed regarding the animal health care sector [3–5]. The aim of this review is to discuss the harmful effects that occupational stress, can potentially have on the psychological well-being of members of the veterinary nursing profession.

Although there has been little research carried out regarding occupational stress in the veterinary nursing profession, available evidence suggests that there are veterinary nurses working in the profession who regard their role as stressful [3, 4]. Whilst occupational stress is not a unique concept relating to members of the human or animal health care sectors, there are unique occupational stressors, such as euthanasia, which members of the animal health care sector are exposed to in comparison to other health care professions, and non-health care workers [3, 4, 6, 7].

Stress within the working environment is not avoidable and therefore labelling stressful events as being the sole reason why an individual might be negatively affected in terms of their health would be inaccurate [8]. It has long been recognised that certain forms of “positive stress” or “eustress”, can result in increased mindful focus on the task in hand, and can be a rewarding experience [9]. An example might be where a veterinary staff member regularly finds themself “under pressure”, but also regularly achieves positive, appreciated outcomes.

On the other hand, when an employee is repeatedly exposed to stress within their working environment and lacks the ability to adaptively cope with it, there is an increasing risk of damage to their psychological well-being. Furthermore, there is a risk of developing occupational stress related conditions such as burnout and or compassion fatigue [10, 11]. Compassion fatigue and burnout are often referred to in the same context, but despite the apparent similarities between the two states, there are different outward expressions of these occupational stress related conditions [12]. The term “compassion fatigue” is viewed as a form of secondary post-traumatic stress disorder, and has been described by the psychotherapist Charles Figley as a “state of tension and preoccupation with the traumatized patients by re-experiencing the traumatic events, avoidance/numbing of reminders persistent arousal (e.g., anxiety) associated with the patient. It is a function of bearing witness to the suffering of others” [13]. “Burnout” is also a psychological response to chronic job stressors, but this response is characterised by Christina Maslach and colleagues as having three main dimensions: “overwhelming exhaustion, feelings of cynicism and detachment from the job, and a sense of ineffectiveness and lack of accomplishment” [10].

How then can one apply the appropriate coping mechanisms to minimise the risk of experiencing burnout and compassion fatigue? Self- care and resilience are two aspects that may promote the use of appropriate coping mechanisms, and therefore the benefits of both will be discussed further within this review. Although there is a lack of readily available evidence in the veterinary literature regarding the negative effects a lack of self-care can have on the well-being of patients, there are reports from other health care professions to suggest that it can cause professional incompetency, ultimately affecting one’s ability to care for their patients [8]. Self-care also has an important role to play in the building and promotion of resilient traits for individuals [14].

Limited research exists in terms of stress-related fatalities in veterinary nursing but there is evidence to suggest that their veterinarian co-workers have an increased incidence of suicide in comparison to other professions [15, 16]. This increased risk of suicide for veterinarians gives further concern regarding the well-being of their nursing counterparts as they are exposed to similar occupational stressors that act as potential triggers for suicide attempts in veterinarians [15, 16]. The worrying statistics that exist for veterinarians in terms of suicide rates give further reasoning for discussing the importance of occupational stress defensive mechanisms in this review.

## Burnout

Burnout has been referred to as an ‘unintentional end point’ for certain individuals who are exposed to chronic stress within their working environment [10]. Burnout, if not managed in an appropriate way can have a negative effect on the mental and physical well-being of an employee, with the possibility of disrupting not only their professional life but also their personal life [17]. Examples of work place stressors that may put an individual at risk from experiencing burnout are long working hours, conflict at work, work overload, high demand low control working environments and working in an environment in which there is little or no social support mechanisms [3, 10, 18–20].

When an employee has reached a stage in which they are suffering from burnout, they may experience one or more of the following: emotional exhaustion, cynicism, and a reduced sense of personal accomplishment in regards to their own work [10, 21, 22]. Although it is possible for an individual to experience all three dimensions when one is suffering from burnout, it is accepted in the literature that feelings of emotional exhaustion is the dimension most associated with this condition and that this can put a person at an increased risk of developing poor mental or physical health [10, 17, 21]. Although exhaustion is the most prevalent of the three dimensions of burnout, it is the degree of exhaustion that can determine maladaptive coping mechanisms such as cynicism and depersonalisation and lead to a lack of self-worth in relation to one’s own personal accomplishments at work [10, 23, 24].

Increased levels of absenteeism at work may be a noticeable sign that an employee is finding it difficult to cope with the demands of the job [25]. When an individual has reached a point within their working environment in which they are feeling extremely exhausted due to occupational related stress, they may be of the opinion that availing of a few “sick days” may aid recuperation [25]. The individual may be using absenteeism as a coping mechanism to help combat the state of exhaustion that they are feeling [25]. The concern with this style of coping is that returning to work may expose the employee to the same stressors as before. Although initially the damage may only be short term on the psychological well-being of an employee, long term damage, not only to the mind but also to physiological processes can put the individual at an increased risk in terms of deteriorating health which may result in long term absenteeism from work [25].

If an employee is experiencing burnout, absenteeism may not be the only noticeable sign. Alternatively, a veterinary staff member may be “present”, but less effective, due to the effects of burnout. The concept of “presenteeism” is used to describe the situation where people continue to turn up for work despite suffering from ill heath such that would normally prompt rest and recuperation [26]. Furthermore, occupational groups whose roles are to provide care or welfare services have a substantially increased risk of being at work when sick [26]. A person may therefore self-identify as “exhausted” due to the demands of the job, but may continue to attend the workplace, however their working standards may decline significantly. Disestel et al. [21] examined the performance of cognitive tasks by nursing home workers, and found a negative association between exhaustion levels and performance capabilities, but it was not until there was a high demand placed on an individual’s cognitive control that this relationship between exhaustion and performance became evident. The implication of this study is that the risk may be increased where individuals are in a role which places high demands on their cognitive control [21]. From this, veterinary staff who are in a state of burnout may not be able to process information and adapt from moment to moment as situations change, such as in a medical emergency.

## Compassion fatigue

In comparison to burnout, compassion fatigue results from bearing witness to, and a need to relieve the suffering of others [11, 27]. When a person is suffering from burnout in a particular job, leaving that job will often be the solution, as it was the work place environment itself that was causing the stress. When a person is suffering from compassion fatigue, it is as a result of an emotional depletion due to the nature of their work, and therefore the solution is more complex [28].

Working in a role that places emotional demands on an employee, such as a need to show compassion and empathy towards clients who are emotionally distressed, puts that person at risk from experiencing compassion fatigue [27]. Veterinary nursing is a caring profession and those who seek a career within this field do so because of a natural desire to care for animals [3]. Individuals who are naturally empathetic towards others are also highly desirable in veterinary nursing or any animal care role, but it is the individuals who are the most caring and empathetic towards others that will be most at risk from experiencing compassion fatigue [11, 27, 28]. The signs of compassion fatigue may be similar to burnout in that absenteeism, presenteeism and a decrease in effectiveness and quality of work may be observed in people who are affected by this condition. In a similar manner as that associated with burnout, the individual may become more cynical and show a lack of compassion towards patients and clients and may become increasingly anti-social both at work and at home [11, 28].

Although compassion fatigue can have detrimental effects on an employee who is experiencing it, it may also affect others in the workplace if there is not a greater emphasis placed on dealing with the problem in an effective way. Dobbs [29] uses the term ‘organisational compassion fatigue’ to describe the trickledown effect that compassion fatigue can have within a workplace environment. This can cause a rift between co-workers and can cause an overall decline in the company’s performance standards. It is therefore important that compassion fatigue is recognised on a personal and organisational level and appropriate steps are taken to combat it [29].

Ensuring that staff are able to recognise the signs of compassion fatigue in each other and are able to engage in healthy coping strategies is important in order to avoid the spread of the condition. For example, healthy coping strategies may include appropriate debriefing sessions among willing participants, particularly after an emotionally stressful encounter with a client (e.g. euthanasia of a pet) [29]. ‘Compassion satisfaction’, a term used by Yoder [27] to describe one’s ability to find happiness in their work, is another area that has been recognised to act as a buffering type mechanism against compassion fatigue and burnout. Aspects of one’s role in veterinary nursing that may provide satisfaction can be an improvement in the health of a patient or gratitude from a client [11].

## Coping strategies

With early indications suggesting that veterinary nursing may be in an ‘at risk’ category for occupational stress [3, 4], why is it that some people are affected more than others? According to Bartram and Gardener [30], the answer may be that what one person finds stressful another person may not. There may be two possible reasons for this. Firstly, the event that one person finds stressful might not be viewed in the same way by another person and secondly, the coping strategies employed by both individuals may vary greatly [30].

Coping strategies employed by people are often described under two headings, adaptive (active) or maladaptive (passive) [30]. Adaptive coping strategies are regarded as being the best for coping with stress in the long term. Adaptive coping strategies aim to positively tackle the stressor or the emotional response to it so that an individual can not only overcome the adverse event but also learn from it which will allow them to cope better if faced with similar situations in the future [30]. Maladaptive coping strategies are regarded as being harmful as they only act to temporarily forget stressful events and or emotional responses to it, therefore not allowing the individual to learn how to deal with similar situations in the future. Examples of maladaptive coping strategies include substance abuse, excessive sleeping patterns, denial of emotions and isolation and avoidance strategies [19, 30].

Coping strategies may also be discussed of in terms of tackling the actual cause of the stress (problem-focused) or tackling the emotional response (emotion-focused) that occur as a result of the stress [17, 30, 31]. There is evidence to suggest that problem-focused coping strategies are more beneficial to people in comparison to emotion-focused strategies [4, 32]. Chang et al. [32] suggest that using emotion-focused coping strategies can actually increase stress levels and have a negative effect on an individual’s well-being. The study carried out by Foster and Maples [4] which involved various members of veterinary support staff, including veterinary nurses, found that individuals who primarily used emotion-focused coping strategies did not feel that they had managed to deal with the adverse event in such a way that they were not negatively affected by it. Bartram and Gardner [30] suggest that using emotion-focused coping strategies is appropriate in certain situations and therefore can also be an adaptive form of coping.

It may be the case that the best form of coping with a stressor is to employ a combination of problem-focused and emotion-focused coping strategies [30]. An individual may need to examine the stressor itself to determine if the impact can be changed while also aiming to normalise their emotional response to it [30]. The issue of euthanasia as a stressor within the veterinary profession may require the individual to employ both problem and emotion-focused coping strategies. For example, improving the veterinary professional’s technique, becoming more skilled and therefore more efficient, may help with reducing some of the stress involved during the process of euthanasia [33]. Recognition and acceptance of one’s emotional response along with talking to others about how they feel are examples of emotion-focused strategies that have reportedly been used by people who are involved in the process of euthanasia [33].

## Organisational culture

The Job Demand-Resources Model of work-related wellbeing attributes the development of workplace strain to an imbalance between (1) job demands such as sustained physical and/or psychological effort, and (2) job resources, including organisational support structures, that assist the worker to achieve their work goals, reduce job demands and facilitate personal growth [34]. This model has been applied to veterinary nursing in New Zealand, in a study which found that lack of workplace resources was associated with emotional exhaustion in this group [35]. Black et al. [3] found organisational support to be a significant factor in predicting job satisfaction among a number of veterinary nurses working in Australia and as discussed previously, finding satisfaction in one’s job may buffer certain stressful events [27]. Building a good team environment acts as a good support mechanism and may include components such as promoting good communication skills among all members of staff, providing support to allow employees to progress in areas of further educational development and also ensuring that all team members are orientated towards the same goals at work [18]. Kimber and Gardner [35] noted that veterinary nurses who perceived their team relationships to be high also perceived job resources to be high. These researchers also identified good strategies for workplace management for veterinary nurses, the provision of opportunities for ongoing learning and professional development, attention to planning and preparation, and appropriate acknowledgement of good work performance.

Debriefing in the workplace can be an important component of stress reduction as it allows for events, such as euthanasia, which are perceived as stressful to be properly appraised [11]. Debriefing is often neglected in veterinary nursing, particularly in the context of euthanasia [11], however appraisal of a stressful event is important as it allows appropriate coping mechanisms to be applied [30].

## Self-care

Taking personal care of one’s own mental and physical health is just as important as taking care of the patient’s health. Mills et al. [36] suggest that a lack of self-care and the ability to be compassionate towards oneself during times of stress will have an effect on one’s ability to provide care and compassion to others. An individual who neglects to self-care is at risk from engaging in maladaptive coping strategies which may impair their ability to work to the standards that are required of them by their profession [8]. For some, improvement in self-care strategies may require certain lifestyle changes such as eating healthily, exercising more or taking up relaxation techniques such as meditation or yoga [11, 37, 38].

Some self-care strategies may not require lifestyle changes but rather a conscious decision to avoid being over-worked within one’s job [39]. In reality many veterinary staff are not in control of their workload, as this is determined by their employer, and can include both normal and “on-call” work. Reduction in sleep hours may have a negative impact on personal performance and team effectiveness and may affect an individual’s health [16, 40], and therefore this may be a matter for the practice manager rather than the individual veterinary staff member.

Mindfulness is an aspect of self-care that has been discussed in terms of the beneficial effects it can have on stress prevention and reduction [41]. Learning to be more compassionate towards oneself has been discussed in psychology literature as being a factor that allows an individual to apply adaptive coping strategies when confronted with a stressful event [42]. Without the ability to be compassionate towards oneself during times of stress an individual may find themselves experiencing what Findlay-Jones et al. [42] describe as ‘emotion regulation difficulties’ and this will only add further intensity to the negative effects of the stressor.

Evidence from other occupations such as human health care nursing suggests that further research into this area would be beneficial, not only to the veterinary nurse but also to the patients that are in their care [43].

It is possible for people to engage in processes that may help to build upon their own levels of self-compassion. One such way to enhance the levels of self-compassion is to engage in training that focuses on the individual becoming more aware of what is happening at a present point in time with the aim of being able to identify a stressor and have the ability to accept the emotions that are connected to it, whether they are positive or negative [44]. This mindfulness-based training can help an individual to avoid negative emotion regulation strategies such as rumination and avoidance training at an early stage (e.g. undergraduate training) may better prepare an individual for the difficulties they may face in their forthcoming careers [41, 44].

## Psychological resilience

There are various definitions within occupational stress related literature that define the term “resilience”. Jackson et al. [45] define it as ‘the ability of an individual to adjust to adversity, maintain equilibrium, retain a sense of control over their environment and continue to move on in a positive manner’. However, not all definitions are in agreement with each other. For example, the Oxford English Dictionary [46] defines resilience as ‘the quality or fact of being able to recover quickly or easily from, or resist being affected by, a misfortune, shock, illness, etc.; robustness; “adaptability”, however Bonano [47] suggests that using the word “recovery” is an inaccurate way of defining resilience. Bonano also suggests that an individual who is resilient will be able to adaptively cope when faced with adversity, therefore not requiring any period of time to recover after the event.

As discussed previously, when confronted with adverse situations the effect it will have on an individual is greatly influenced by the style of coping in which they employ to tackle the event. The importance of resilience for individuals working in potentially stressful occupations is that it allows for an individual to appropriately appraise a stressful situation which in turn will promote an adaptive rather that maladaptive style of coping [48, 49].. Mealer et al. [50] provide evidence to support the claim that resilience is beneficial to people that work in potentially stressful occupations. Their study involved human health care nurses working in an intensive care setting, and found that nurses who scored higher on a defined “resiliency” scale were less likely to be affected by occupational stress and as a result there was a significantly reduced risk of experiencing stress related conditions such as burnout.

In veterinary literature on resilience, Cake et al. identify “emotional competence” as given particular prominence, describing a high level of emotional processing capabilities required for success [51]. Characteristics of resilience are generally described as personal qualities or traits such as optimism, self-confidence, level headedness, hardiness, and having the ability to be resourceful during times of adversity [45, 50, 52]. A person who is described as being “hardy” is one that will always commit to the task regardless of the perceived difficulty it presents with, will feel they are always in control of the task and will view any unforeseen changes as a challenge and something to overcome rather than a negative experience and a reason to give up [49, 52]. As mentioned previously, engaging in healthy self-care strategies is an area that can be beneficial to individuals in terms promoting these resilient traits [14]. It is important, however, that we do not only think of resilience in terms of it being a personality trait which is already present in people, but as a skill that may be improved upon over time.

## Resilience building

Individuals may become more, “hardy” and resilient if they partake in educational training that focuses on resilience building [49, 52]. Although there appears to be no “gold standard” for resilience training [53], there are examples within the professional veterinary and human nursing literature which point to improvement in personal resilience following such training. In one such study in nurses [49], participants undertook a training module which focused on occupational stress, mindfulness, hardiness, and how to apply adaptive styles of coping during times of adversity. Although the sample size was small, participants of the study did appear to have increased levels of hardiness upon completion of the module. Veterinary examples are limited, but include a recent example of resilience training in undergraduate veterinary students [54]. Again, although a small, single group study with no control group due to ethical limitations, the results indicate that appropriate training in resilience-building strategies can potentially support the development of a more resilient approach in veterinary professional’s personal life and career.

## Conclusion

Although evidence is relatively limited regarding occupational stress which is directly related to the veterinary nursing profession, this review has aimed at providing veterinary professionals, and in particular, veterinary nurses with a clearer understanding of the detrimental effects it can potentially have on an individual’s well-being if they are unable to utilise the appropriate style of coping mechanisms. Burnout and compassion fatigue are two occupational stress related conditions that veterinary professionals are at risk of experiencing. By providing information regarding burnout and compassion fatigue and what actions one may take in terms of safe guarding one’s psychological well-being, the hope is that this will promote the use of adaptive styles of coping for veterinary professionals when they are faced with adversity throughout their careers. Self-care and resilience have been discussed in terms of how they promote the use of adaptive coping mechanisms. It is difficult to discuss self-care and resilience in isolation as they often work in tandem with each other, i.e. engaging in self-care will ultimately promote a more resilient individual.

The current literature suggests that there should be a greater emphasis placed on awareness of self-care and occupational stress during undergraduate education. It may be the case that due to a lack of readily available evidence from within the veterinary nursing profession as to the negative effects that work related stress can have on its members, the process of introducing learning opportunities in occupational stress, self-care and resilience into the undergraduate graduate curriculum may not be of high priority for academics. As veterinary nurses are in an at-risk category in terms of occupational stress, and early intervention may prove to be beneficial. Further research into the area of occupational stress is recommended for this cohort of veterinary professionals.
